# Bis(4-carboxy­piperidinium) 5-nitro­isophthalate

**DOI:** 10.1107/S1600536810017927

**Published:** 2010-05-19

**Authors:** Na Li

**Affiliations:** a College of Chemistry, Tianjin Key Laboratory of Structure and Performance for Functional Molecule, Tianjin Normal University, Tianjin 300387, People’s Republic of China

## Abstract

Cocrystallization of 4-carboxypiperdine with 5-nitro­isophthalic acid afforded the title salt, 2C_6_H_12N_O_2_^+^·C_8_H_3_NO_6_^2−^, in which the heterocyclic N atoms are protonated and the carboxylic acid groups are deprotonated. In the crystal, inter­molecular N—H⋯O and O—H⋯O hydrogen-bonding inter­actions assemble the ions into a three-dimensional network.

## Related literature

For mol­ecular self-assembly by non-covalent inter­actions and its potential applications, see: Remenar *et al.* (2003 ▶); Oxtoby *et al.* (2005 ▶); Zaworotko (2001 ▶); Wang *et al.* (2009 ▶). For 4-piperdinecarboxylic acid as a zwitterion in aqueous solution, see: Mora *et al.* (2002 ▶) and for its ability to act selectively as a bridging or terminal ligand, see: Inomata *et al.* (2002 ▶). For related structures, see: Adams *et al.* (2006 ▶); Podesta & Orpen (2002 ▶); Delgado *et al.* (2001 ▶); Zhang *et al.* (2009 ▶).
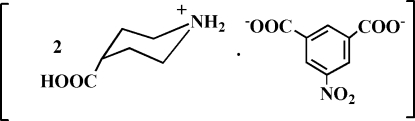


## Experimental

### 

#### Crystal data

2C_6_H_12_NO_2_^+^·C_8_H_3_NO_6_^2−^
*M* *_r_* = 469.45
Monoclinic, 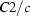

*a* = 23.6865 (12) Å
*b* = 8.2478 (4) Å
*c* = 22.5140 (11) Å
β = 92.396 (1)°
*V* = 4394.5 (4) Å^3^
*Z* = 8
Mo *K*α radiation
μ = 0.12 mm^−1^
*T* = 296 K
0.25 × 0.24 × 0.20 mm

#### Data collection

Bruker APEXII CCD area-detector diffractometer
Absorption correction: multi-scan (*SADABS*; Sheldrick, 1996 ▶) *T*_min_ = 0.972, *T*_max_ = 0.977
10813 measured reflections
3855 independent reflections
3272 reflections with *I* > 2σ(*I*)
*R*_int_ = 0.016

#### Refinement

*R*[*F* ^2^ > 2σ(*F* ^2^)] = 0.039
*wR*(*F* ^2^) = 0.101
*S* = 1.04
3855 reflections
300 parameters
H-atom parameters constrained
Δρ_max_ = 0.30 e Å^−3^
Δρ_min_ = −0.23 e Å^−3^

### 

Data collection: *APEX2* (Bruker, 2003 ▶); cell refinement: *SAINT* (Bruker, 2001 ▶); data reduction: *SAINT*; program(s) used to solve structure: *SHELXS97* (Sheldrick, 2008 ▶); program(s) used to refine structure: *SHELXL97* (Sheldrick, 2008 ▶); molecular graphics: *SHELXTL* (Sheldrick, 2008 ▶) and *DIAMOND* (Brandenburg & Berndt, 1999 ▶); software used to prepare material for publication: *SHELXL97*.

## Supplementary Material

Crystal structure: contains datablocks I, global. DOI: 10.1107/S1600536810017927/bt5271sup1.cif

Structure factors: contains datablocks I. DOI: 10.1107/S1600536810017927/bt5271Isup2.hkl

Additional supplementary materials:  crystallographic information; 3D view; checkCIF report

## Figures and Tables

**Table 1:** Hydrogen-bond geometry (Å, °)

*D*—H⋯*A*	*D*—H	H⋯*A*	*D*⋯*A*	*D*—H⋯*A*
O7—H7⋯O2^i^	0.82	1.72	2.5204 (16)	166
O9—H9⋯O3^ii^	0.82	1.75	2.5495 (17)	164
N2—H2*A*⋯O4^iii^	0.90	1.98	2.8629 (19)	166
N2—H2*B*⋯O4^iv^	0.90	2.01	2.7823 (17)	143
N3—H3*A*⋯O1^v^	0.90	1.83	2.7220 (18)	171
N3—H3*B*⋯O8^vi^	0.90	1.89	2.755 (2)	161

Symmetry codes: (i) 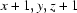; (ii) 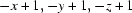; (iii) 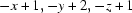; (iv) 
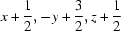
; (v) 
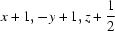
; (vi) 
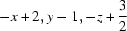
.

**Fig. 1.**
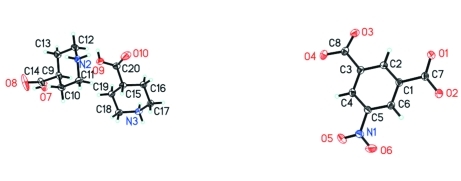
The asymmetric unit of the title complex. Displacement ellipsoids are drawn at the 30% probability level.

**Fig. 2.**
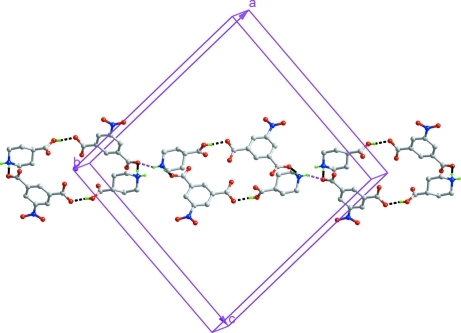
Partial packing diagram of the title compound. H bonds drawn as dashed lines.

## supplementary crystallographic information

### Comment

Recently, molecular self-assembly by non-covalent interactions has attacted considerable interest in supramolecular chemistry and crystal engineering fields due to its potential applications in materials (Zaworotko, 2001), molecular recognition (Wang *et al., *2009; Oxtoby *et al.*, 2005), and pharmaceutical chemistry (Remenar *et al., *2003). Obviously, the conguated organic components with rich carboxylate or amino groups have became good blocks for the construction of self-assembly systems, since popular hydrogen-bonding and π··· π interactions are the main driven forces of the assembly process. In this regard, bearing two functional groups (–NH– and –COOH–) capable of producing abundant hydrogen-bonding interactions as well as coordination with transitional ions, 4-piperdinecarboxylic acid (Hpipe) exists as a zwitterion with the amino group protonated and the carboxylic group deprotonated in aqueous solution (Mora *et al.*, 2002). While, in the solid state, the zwitterionic Hpipe can either coordinate with metal ions by its deprotonated carboxylate group or form cocrystals with other compensated components by hydrogen-bonding interactions (Inomata *et al.* 2002; Adams *et al.*, 2006; Podesta & Orpen, 2002; Zhang *et al.*, 2009; Delgado *et al.* 2001). To continue to investigate the self-assembly behavior of Hpipe in the solid state, herein, we report the cocrystal of Hpipe and 5-nitroisophthalic acid (H_2_nip).

As shown in Figure 1, the asymmetric unit of (I) comprises one doubly deprotonated 5-nitro-isophthalate anion (nip^2-^) and two chemically equal but crystallographically independent 4-piperdinecarboxylic acid cations (H_2_pipe^+^). In the crystal, a pair of symmetry-related nip anions and two crystallographically equivalent Hpipe^+^ cations are connected together in a head-to-tail manner by N–H···O and O–H···O hydrogen-bonds between the protonated amino/carboxylic groups of H_2_pipe^+ ^and the deprotonated carboxylate of nip anions (Table 1). Thus, closed four-component-based supramolecular rings are gerenated and extended in [1 -1 1] direction (Figure 2). Then, these supramolecular rings are further non-covalently extended by pairs of the second crystallographically unique Hpipe^+^ cation, leading to a three-dimensional (3-D) hdrogen-bonds network (Figure 3 and Table 1). Thus, the abundant hydrogen-bonding interactions significantly dominate the formation of 3-D supramolecular network of the title cocrystal.

### Experimental

4-Piperidinecarboxylic acid (0.1 mmol, 12.9 mg) and 5–nitroisophthalic acid (0.1 mmol, 21.0 mg) were dissolved in a mixed CH_3_OH—H_2_O solution (v : v = 5 : 2, 7.0 ml) and stirred constantly for about 30 min. The resulting mixture was then filtered. Colorless block-shaped crystals suitable for X–ray diffraction were collected by slow evaporation of the filtrate in one week. Yield: 65% based on 4-piperdinecarboxylic acid. Anal. calcd for C_20_H_27_N_3_O_10_: C, 51.17; H, 5.80; N, 8.95%. Found: C, 51.27; H, 5.91; N, 9.03%.

### Refinement

H atoms were located in difference maps, but were subsequently placed in calculated positions and treated as riding, with C – H = 0.93 (for methylene) or 0.97 (for aromatic C – H), O – H = 0.82, and N – H = 0.90 Å. All H atoms were allocated displacement parameters related to those of their parent atoms [Uiso(H) = 1.2 Ueq(C, N) or 1.5 Ueq(O)].

### Crystal data

**Table:**

2C_6_H_12_NO_2_^+^·C_8_H_3_NO_6_^2^^−^	*F*(000) = 1984
*M**_r_* = 469.45	*D*_x_ = 1.419 Mg m^−^^3^
Monoclinic, *C*2/*c*	Mo *K*α radiation, λ = 0.71073 Å
*a* = 23.6865 (12) Å	Cell parameters from 5783 reflections
*b* = 8.2478 (4) Å	θ = 2.6–27.7°
*c* = 22.5140 (11) Å	µ = 0.12 mm^−^^1^
β = 92.396 (1)°	*T* = 296 K
*V* = 4394.5 (4) Å^3^	Block, colourless
*Z* = 8	0.25 × 0.24 × 0.20 mm

### Data collection

**Table:**

Bruker APEXII CCD area-detector diffractometer	3855 independent reflections
Radiation source: fine-focus sealed tube	3272 reflections with *I* > 2σ(*I*)
graphite	*R*_int_ = 0.016
phi and ω scans	θ_max_ = 25.0°, θ_min_ = 1.7°
Absorption correction: multi-scan (*SADABS*; Sheldrick, 1996)	*h* = −26→28
*T*_min_ = 0.972, *T*_max_ = 0.977	*k* = −6→9
10813 measured reflections	*l* = −26→25

### Refinement

**Table:**

Refinement on *F*^2^	Primary atom site location: structure-invariant direct methods
Least-squares matrix: full	Secondary atom site location: difference Fourier map
*R*[*F*^2^ > 2σ(*F*^2^)] = 0.039	Hydrogen site location: inferred from neighbouring sites
*wR*(*F*^2^) = 0.101	H-atom parameters constrained
*S* = 1.04	*w* = 1/[σ^2^(*F*_o_^2^) + (0.0446*P*)^2^ + 3.6101*P*] where *P* = (*F*_o_^2^ + 2*F*_c_^2^)/3
3855 reflections	(Δ/σ)_max_ = 0.001
300 parameters	Δρ_max_ = 0.30 e Å^−^^3^
0 restraints	Δρ_min_ = −0.23 e Å^−^^3^

### Special details

**Table:**

Geometry. All esds (except the esd in the dihedral angle between two l.s. planes) are estimated using the full covariance matrix. The cell esds are taken into account individually in the estimation of esds in distances, angles and torsion angles; correlations between esds in cell parameters are only used when they are defined by crystal symmetry. An approximate (isotropic) treatment of cell esds is used for estimating esds involving l.s. planes.
Refinement. Refinement of *F*^2^ against ALL reflections. The weighted *R*-factor wR and goodness of fit *S* are based on *F*^2^, conventional *R*-factors *R* are based on *F*, with *F* set to zero for negative *F*^2^. The threshold expression of *F*^2^ > σ(*F*^2^) is used only for calculating *R*-factors(gt) etc. and is not relevant to the choice of reflections for refinement. *R*-factors based on *F*^2^ are statistically about twice as large as those based on *F*, and *R*- factors based on ALL data will be even larger.

### Fractional atomic coordinates and isotropic or equivalent isotropic displacement parameters (Å^2^)

**Table:**

	*x*	*y*	*z*	*U*_iso_*/*U*_eq_	
O1	0.05079 (5)	0.70916 (18)	−0.03056 (5)	0.0496 (3)	
O2	0.08990 (5)	0.83618 (18)	−0.10541 (5)	0.0508 (3)	
O3	0.16282 (6)	0.63183 (16)	0.15592 (5)	0.0497 (3)	
O4	0.22886 (5)	0.81433 (15)	0.18158 (5)	0.0431 (3)	
O5	0.31864 (6)	1.0750 (2)	0.00708 (7)	0.0634 (4)	
O6	0.26236 (6)	1.1471 (2)	−0.06513 (7)	0.0702 (5)	
O7	1.00128 (5)	0.77960 (18)	0.83217 (6)	0.0561 (4)	
H7	1.0273	0.8104	0.8546	0.084*	
O8	0.96201 (7)	1.0009 (2)	0.86340 (11)	0.1136 (9)	
O9	0.89526 (5)	0.40628 (18)	0.75229 (5)	0.0520 (4)	
H9	0.8714	0.3961	0.7774	0.078*	
O10	0.82217 (6)	0.3599 (3)	0.69185 (7)	0.0915 (7)	
N1	0.27300 (6)	1.07054 (18)	−0.01985 (7)	0.0410 (3)	
N2	0.78899 (5)	0.87733 (17)	0.76390 (6)	0.0333 (3)	
H2A	0.7801	0.9650	0.7854	0.040*	
H2B	0.7578	0.8468	0.7426	0.040*	
N3	0.99531 (6)	0.26082 (18)	0.57233 (6)	0.0410 (3)	
H3A	1.0170	0.2687	0.5406	0.049*	
H3B	1.0025	0.1645	0.5898	0.049*	
C1	0.14080 (6)	0.83039 (19)	−0.01316 (7)	0.0310 (3)	
C2	0.14657 (6)	0.77035 (19)	0.04446 (7)	0.0314 (3)	
H2	0.1191	0.7006	0.0582	0.038*	
C3	0.19210 (6)	0.81163 (19)	0.08202 (6)	0.0304 (3)	
C4	0.23391 (6)	0.9130 (2)	0.06152 (7)	0.0324 (4)	
H4	0.2646	0.9434	0.0861	0.039*	
C5	0.22846 (6)	0.96748 (19)	0.00345 (7)	0.0314 (3)	
C6	0.18275 (6)	0.92973 (19)	−0.03393 (7)	0.0326 (3)	
H6	0.1801	0.9704	−0.0725	0.039*	
C7	0.08946 (6)	0.7881 (2)	−0.05230 (7)	0.0350 (4)	
C8	0.19502 (7)	0.7475 (2)	0.14504 (7)	0.0335 (4)	
C9	0.90789 (6)	0.8298 (2)	0.79810 (8)	0.0386 (4)	
H9A	0.9167	0.7345	0.7742	0.046*	
C10	0.88790 (7)	0.9662 (2)	0.75661 (8)	0.0424 (4)	
H10A	0.8819	1.0635	0.7797	0.051*	
H10B	0.9170	0.9894	0.7288	0.051*	
C11	0.83367 (7)	0.9221 (2)	0.72253 (8)	0.0426 (4)	
H11A	0.8210	1.0135	0.6983	0.051*	
H11B	0.8405	0.8317	0.6962	0.051*	
C12	0.80657 (7)	0.7439 (2)	0.80488 (8)	0.0411 (4)	
H12A	0.8121	0.6456	0.7822	0.049*	
H12B	0.7769	0.7236	0.8323	0.049*	
C13	0.86074 (7)	0.7858 (2)	0.83956 (8)	0.0422 (4)	
H13A	0.8725	0.6940	0.8640	0.051*	
H13B	0.8540	0.8766	0.8657	0.051*	
C14	0.96009 (7)	0.8790 (2)	0.83435 (9)	0.0433 (4)	
C15	0.91160 (7)	0.3924 (2)	0.64910 (7)	0.0358 (4)	
H15	0.9051	0.4959	0.6286	0.043*	
C16	0.89746 (7)	0.2580 (2)	0.60467 (7)	0.0405 (4)	
H16A	0.9024	0.1536	0.6240	0.049*	
H16B	0.8582	0.2674	0.5910	0.049*	
C17	0.93479 (7)	0.2668 (2)	0.55192 (8)	0.0436 (4)	
H17A	0.9264	0.1766	0.5253	0.052*	
H17B	0.9273	0.3666	0.5302	0.052*	
C18	1.01064 (7)	0.3933 (2)	0.61503 (8)	0.0448 (4)	
H18A	1.0056	0.4974	0.5955	0.054*	
H18B	1.0501	0.3830	0.6280	0.054*	
C19	0.97391 (7)	0.3853 (2)	0.66851 (8)	0.0418 (4)	
H19A	0.9830	0.4754	0.6949	0.050*	
H19B	0.9815	0.2855	0.6901	0.050*	
C20	0.87172 (7)	0.3847 (2)	0.69963 (8)	0.0406 (4)	

### Atomic displacement parameters (Å^2^)

**Table:**

	*U*^11^	*U*^22^	*U*^33^	*U*^12^	*U*^13^	*U*^23^
O1	0.0375 (7)	0.0741 (9)	0.0371 (7)	−0.0206 (6)	0.0007 (5)	−0.0047 (6)
O2	0.0380 (7)	0.0776 (10)	0.0356 (7)	−0.0124 (6)	−0.0110 (5)	0.0119 (6)
O3	0.0657 (8)	0.0494 (8)	0.0343 (6)	−0.0115 (7)	0.0051 (6)	0.0055 (6)
O4	0.0462 (7)	0.0512 (7)	0.0311 (6)	0.0052 (6)	−0.0100 (5)	0.0004 (5)
O5	0.0413 (8)	0.0816 (11)	0.0667 (9)	−0.0263 (7)	−0.0037 (7)	−0.0034 (8)
O6	0.0663 (10)	0.0886 (12)	0.0559 (9)	−0.0231 (8)	0.0044 (7)	0.0300 (8)
O7	0.0410 (7)	0.0657 (9)	0.0596 (9)	0.0117 (7)	−0.0218 (6)	−0.0146 (7)
O8	0.0655 (11)	0.0722 (12)	0.198 (2)	0.0142 (9)	−0.0592 (13)	−0.0777 (14)
O9	0.0445 (7)	0.0722 (9)	0.0398 (7)	−0.0102 (7)	0.0066 (6)	−0.0009 (7)
O10	0.0310 (8)	0.191 (2)	0.0526 (9)	−0.0075 (10)	0.0066 (6)	−0.0059 (11)
N1	0.0385 (8)	0.0437 (8)	0.0414 (8)	−0.0086 (6)	0.0070 (6)	−0.0033 (7)
N2	0.0262 (6)	0.0404 (8)	0.0328 (7)	−0.0002 (6)	−0.0040 (5)	−0.0028 (6)
N3	0.0386 (8)	0.0470 (9)	0.0379 (8)	0.0116 (6)	0.0060 (6)	0.0081 (7)
C1	0.0268 (7)	0.0360 (9)	0.0301 (8)	0.0001 (6)	−0.0015 (6)	−0.0016 (7)
C2	0.0283 (8)	0.0356 (9)	0.0305 (8)	−0.0024 (6)	0.0026 (6)	−0.0003 (7)
C3	0.0302 (8)	0.0342 (8)	0.0268 (7)	0.0035 (6)	0.0008 (6)	−0.0020 (6)
C4	0.0278 (8)	0.0370 (9)	0.0320 (8)	0.0006 (6)	−0.0041 (6)	−0.0049 (7)
C5	0.0294 (8)	0.0321 (8)	0.0329 (8)	−0.0026 (6)	0.0024 (6)	−0.0015 (7)
C6	0.0325 (8)	0.0372 (9)	0.0278 (8)	0.0023 (7)	−0.0004 (6)	0.0022 (7)
C7	0.0298 (8)	0.0441 (10)	0.0308 (8)	−0.0005 (7)	−0.0018 (6)	−0.0029 (7)
C8	0.0350 (8)	0.0373 (9)	0.0282 (8)	0.0077 (7)	−0.0007 (7)	−0.0014 (7)
C9	0.0287 (8)	0.0333 (9)	0.0532 (10)	0.0005 (7)	−0.0052 (7)	−0.0104 (8)
C10	0.0339 (9)	0.0461 (10)	0.0475 (10)	−0.0069 (8)	0.0057 (7)	0.0022 (8)
C11	0.0378 (9)	0.0546 (11)	0.0353 (9)	−0.0019 (8)	0.0032 (7)	0.0060 (8)
C12	0.0329 (9)	0.0461 (10)	0.0438 (9)	−0.0079 (7)	−0.0041 (7)	0.0103 (8)
C13	0.0375 (9)	0.0449 (10)	0.0433 (10)	−0.0045 (8)	−0.0096 (7)	0.0089 (8)
C14	0.0325 (9)	0.0369 (10)	0.0597 (11)	−0.0035 (7)	−0.0073 (8)	−0.0068 (9)
C15	0.0326 (8)	0.0371 (9)	0.0380 (9)	0.0023 (7)	0.0030 (7)	0.0068 (7)
C16	0.0319 (9)	0.0490 (10)	0.0403 (9)	−0.0024 (7)	−0.0016 (7)	0.0028 (8)
C17	0.0399 (9)	0.0539 (11)	0.0366 (9)	0.0026 (8)	−0.0026 (7)	−0.0001 (8)
C18	0.0303 (9)	0.0591 (12)	0.0453 (10)	−0.0017 (8)	0.0031 (7)	0.0017 (9)
C19	0.0326 (9)	0.0549 (11)	0.0379 (9)	−0.0021 (8)	0.0011 (7)	−0.0015 (8)
C20	0.0329 (9)	0.0459 (10)	0.0431 (10)	0.0039 (7)	0.0035 (7)	0.0036 (8)

### Geometric parameters (Å, °)

**Table:**

O1—C7	1.241 (2)	C5—C6	1.379 (2)
O2—C7	1.260 (2)	C6—H6	0.9300
O3—C8	1.252 (2)	C9—C14	1.508 (2)
O4—C8	1.2524 (19)	C9—C10	1.525 (2)
O5—N1	1.2177 (19)	C9—C13	1.529 (2)
O6—N1	1.216 (2)	C9—H9A	0.9800
O7—C14	1.277 (2)	C10—C11	1.513 (2)
O7—H7	0.8200	C10—H10A	0.9700
O8—C14	1.199 (2)	C10—H10B	0.9700
O9—C20	1.301 (2)	C11—H11A	0.9700
O9—H9	0.8200	C11—H11B	0.9700
O10—C20	1.197 (2)	C12—C13	1.514 (2)
N1—C5	1.469 (2)	C12—H12A	0.9700
N2—C12	1.485 (2)	C12—H12B	0.9700
N2—C11	1.485 (2)	C13—H13A	0.9700
N2—H2A	0.9000	C13—H13B	0.9700
N2—H2B	0.9000	C15—C20	1.510 (2)
N3—C17	1.488 (2)	C15—C16	1.521 (2)
N3—C18	1.490 (2)	C15—C19	1.523 (2)
N3—H3A	0.9000	C15—H15	0.9800
N3—H3B	0.9000	C16—C17	1.512 (2)
C1—C6	1.384 (2)	C16—H16A	0.9700
C1—C2	1.390 (2)	C16—H16B	0.9700
C1—C7	1.512 (2)	C17—H17A	0.9700
C2—C3	1.385 (2)	C17—H17B	0.9700
C2—H2	0.9300	C18—C19	1.516 (2)
C3—C4	1.390 (2)	C18—H18A	0.9700
C3—C8	1.513 (2)	C18—H18B	0.9700
C4—C5	1.383 (2)	C19—H19A	0.9700
C4—H4	0.9300	C19—H19B	0.9700
			
C14—O7—H7	109.5	N2—C11—H11A	109.5
C20—O9—H9	109.5	C10—C11—H11A	109.5
O6—N1—O5	123.39 (15)	N2—C11—H11B	109.5
O6—N1—C5	118.22 (14)	C10—C11—H11B	109.5
O5—N1—C5	118.38 (15)	H11A—C11—H11B	108.1
C12—N2—C11	112.66 (13)	N2—C12—C13	111.15 (14)
C12—N2—H2A	109.1	N2—C12—H12A	109.4
C11—N2—H2A	109.1	C13—C12—H12A	109.4
C12—N2—H2B	109.1	N2—C12—H12B	109.4
C11—N2—H2B	109.1	C13—C12—H12B	109.4
H2A—N2—H2B	107.8	H12A—C12—H12B	108.0
C17—N3—C18	112.38 (13)	C12—C13—C9	111.37 (14)
C17—N3—H3A	109.1	C12—C13—H13A	109.4
C18—N3—H3A	109.1	C9—C13—H13A	109.4
C17—N3—H3B	109.1	C12—C13—H13B	109.4
C18—N3—H3B	109.1	C9—C13—H13B	109.4
H3A—N3—H3B	107.9	H13A—C13—H13B	108.0
C6—C1—C2	118.83 (14)	O8—C14—O7	123.21 (17)
C6—C1—C7	120.71 (14)	O8—C14—C9	122.15 (17)
C2—C1—C7	120.46 (14)	O7—C14—C9	114.63 (15)
C3—C2—C1	121.73 (14)	C20—C15—C16	109.74 (14)
C3—C2—H2	119.1	C20—C15—C19	114.31 (14)
C1—C2—H2	119.1	C16—C15—C19	110.20 (14)
C2—C3—C4	119.53 (14)	C20—C15—H15	107.4
C2—C3—C8	119.36 (14)	C16—C15—H15	107.4
C4—C3—C8	121.11 (14)	C19—C15—H15	107.4
C5—C4—C3	118.01 (14)	C17—C16—C15	111.22 (14)
C5—C4—H4	121.0	C17—C16—H16A	109.4
C3—C4—H4	121.0	C15—C16—H16A	109.4
C6—C5—C4	122.91 (14)	C17—C16—H16B	109.4
C6—C5—N1	118.04 (14)	C15—C16—H16B	109.4
C4—C5—N1	119.05 (14)	H16A—C16—H16B	108.0
C5—C6—C1	118.94 (14)	N3—C17—C16	110.08 (14)
C5—C6—H6	120.5	N3—C17—H17A	109.6
C1—C6—H6	120.5	C16—C17—H17A	109.6
O1—C7—O2	125.10 (15)	N3—C17—H17B	109.6
O1—C7—C1	118.68 (14)	C16—C17—H17B	109.6
O2—C7—C1	116.22 (14)	H17A—C17—H17B	108.2
O3—C8—O4	125.83 (15)	N3—C18—C19	110.39 (14)
O3—C8—C3	116.47 (14)	N3—C18—H18A	109.6
O4—C8—C3	117.70 (15)	C19—C18—H18A	109.6
C14—C9—C10	111.07 (14)	N3—C18—H18B	109.6
C14—C9—C13	109.67 (15)	C19—C18—H18B	109.6
C10—C9—C13	109.44 (13)	H18A—C18—H18B	108.1
C14—C9—H9A	108.9	C18—C19—C15	110.60 (14)
C10—C9—H9A	108.9	C18—C19—H19A	109.5
C13—C9—H9A	108.9	C15—C19—H19A	109.5
C11—C10—C9	111.62 (14)	C18—C19—H19B	109.5
C11—C10—H10A	109.3	C15—C19—H19B	109.5
C9—C10—H10A	109.3	H19A—C19—H19B	108.1
C11—C10—H10B	109.3	O10—C20—O9	122.47 (17)
C9—C10—H10B	109.3	O10—C20—C15	122.50 (16)
H10A—C10—H10B	108.0	O9—C20—C15	115.03 (14)
N2—C11—C10	110.73 (14)		
			
C6—C1—C2—C3	2.2 (2)	C14—C9—C10—C11	−176.73 (15)
C7—C1—C2—C3	−177.66 (14)	C13—C9—C10—C11	−55.51 (19)
C1—C2—C3—C4	−1.3 (2)	C12—N2—C11—C10	−55.94 (19)
C1—C2—C3—C8	177.74 (14)	C9—C10—C11—N2	56.0 (2)
C2—C3—C4—C5	−0.8 (2)	C11—N2—C12—C13	55.84 (19)
C8—C3—C4—C5	−179.84 (14)	N2—C12—C13—C9	−55.4 (2)
C3—C4—C5—C6	2.1 (2)	C14—C9—C13—C12	177.09 (15)
C3—C4—C5—N1	−178.06 (14)	C10—C9—C13—C12	55.02 (19)
O6—N1—C5—C6	16.3 (2)	C10—C9—C14—O8	53.3 (3)
O5—N1—C5—C6	−163.70 (16)	C13—C9—C14—O8	−67.8 (3)
O6—N1—C5—C4	−163.54 (17)	C10—C9—C14—O7	−127.69 (18)
O5—N1—C5—C4	16.5 (2)	C13—C9—C14—O7	111.21 (18)
C4—C5—C6—C1	−1.3 (2)	C20—C15—C16—C17	177.29 (14)
N1—C5—C6—C1	178.86 (14)	C19—C15—C16—C17	−55.98 (19)
C2—C1—C6—C5	−0.8 (2)	C18—N3—C17—C16	−57.40 (19)
C7—C1—C6—C5	178.99 (14)	C15—C16—C17—N3	56.3 (2)
C6—C1—C7—O1	−174.49 (16)	C17—N3—C18—C19	57.68 (19)
C2—C1—C7—O1	5.3 (2)	N3—C18—C19—C15	−56.3 (2)
C6—C1—C7—O2	6.0 (2)	C20—C15—C19—C18	179.86 (15)
C2—C1—C7—O2	−174.16 (15)	C16—C15—C19—C18	55.7 (2)
C2—C3—C8—O3	15.5 (2)	C16—C15—C20—O10	−42.0 (3)
C4—C3—C8—O3	−165.40 (15)	C19—C15—C20—O10	−166.3 (2)
C2—C3—C8—O4	−164.12 (15)	C16—C15—C20—O9	137.65 (16)
C4—C3—C8—O4	15.0 (2)	C19—C15—C20—O9	13.3 (2)

### Hydrogen-bond geometry (Å, °)

**Table:**

*D*—H···*A*	*D*—H	H···*A*	*D*···*A*	*D*—H···*A*
O7—H7···O2^i^	0.82	1.72	2.5204 (16)	166
O9—H9···O3^ii^	0.82	1.75	2.5495 (17)	164
N2—H2A···O4^iii^	0.90	1.98	2.8629 (19)	166
N2—H2B···O4^iv^	0.90	2.01	2.7823 (17)	143
N3—H3A···O1^v^	0.90	1.83	2.7220 (18)	171
N3—H3B···O8^vi^	0.90	1.89	2.755 (2)	161

Symmetry codes: (i) *x*+1, *y*, *z*+1; (ii) −*x*+1, −*y*+1, −*z*+1; (iii) −*x*+1, −*y*+2, −*z*+1; (iv) *x*+1/2, −*y*+3/2, *z*+1/2; (v) *x*+1, −*y*+1, *z*+1/2; (vi) −*x*+2, *y*−1, −*z*+3/2.

## References

[bb1] Adams, C. J., Crawford, P. C., Orpen, A. G. & Podesta, T. J. (2006). *Dalton Trans* pp. 4078–4092. 10.1039/b604319d 16924286

[bb2] Brandenburg, K. & Berndt, M. (1999). *DIAMOND* Crystal Impact GbR, Bonn, Germany.

[bb3] Bruker (2001). *SAINT* Bruker AXS Inc., Madison, Wisconsin, USA.

[bb4] Bruker (2003). *APEX2* Bruker AXS Inc., Madison, Wisconsin, USA.

[bb5] Delgado, G., Mora, A. J. & Bahsas, A. (2001). *Acta Cryst.* C**57**, 965–967. 10.1107/s0108270101007958 11498628

[bb6] Inomata, Y., Ando, M. & Howell, F. S. (2002). *J. Mol. Struct.* **616**, 201–212.

[bb7] Mora, A. J., Delgado, G., Ramírez, B. M., Rincón, L., Almeida, R., Cuervo, J. & Bahsas, A. (2002). *J. Mol. Struct* **615**, 201–208.

[bb8] Oxtoby, N. S., Blake, A. J., Champness, N. R. & Wilson, C. (2005). *Chem. Eur. J* **11**, 1–13.

[bb9] Podesta, T. J. & Orpen, A. G. (2002). *CrystEngComm*, **4**, 336–342.

[bb10] Remenar, J. F., Morissette, S. L., Peterson, M. L., Moulton, B., MacPhee, J. M., Guzmán, H. R. & Almarsson, Ö. (2003). *J. Am. Chem. Soc* **125**, 8456–8457. 10.1021/ja035776p 12848550

[bb11] Sheldrick, G. M. (1996). *SADABS* University of Göttingen, Germany.

[bb12] Sheldrick, G. M. (2008). *Acta Cryst.* A**64**, 112–122. 10.1107/S0108767307043930 18156677

[bb13] Wang, L.-L., Chang, H. & Yang, E.-C. (2009). *Acta Cryst.* C**65**, o492–o494. 10.1107/S010827010903340X 19726869

[bb14] Zaworotko, M. J. (2001). *Chem. Commun* pp. 1–9.

[bb15] Zhang, R.-W., Wang, L.-L. & Zhao, X.-J. (2009). *Acta Cryst.* E**65**, m664–m665. 10.1107/S160053680901811X PMC2969669 21583026

